# Primary Angiosarcoma of the Breast after Bilateral Breast Reduction

**DOI:** 10.1155/2018/7390987

**Published:** 2018-06-07

**Authors:** Justus Philip, Elizabeth Bender, Kristy Waite

**Affiliations:** ^1^Department of Surgery, Summa Health System Akron City Hospital, 900 W. Market St., Apt. No. 400, Akron, OH 44313, USA; ^2^Department of Surgery, Summa Health System Akron City Hospital, 75 Arch St. Suite 406, Akron, OH 44304, USA; ^3^Department of Pathology, Summa Health System Akron City Hospital, 525 E. Market St., Akron, OH 44304, USA

## Abstract

Angiosarcoma of the breast is a rare malignancy of endothelial cell origin, representing less than 1% of all breast malignancy. Primary angiosarcomas can occur in the setting of chronic lymphedema, but it also may occur spontaneously without any preceding treatment. Surgery is the primary therapeutic intervention for breast angiosarcomas with radiation and chemotherapy as adjuvant treatment. Angiosarcomas are aggressive and tend to have a high risk of local and metastatic recurrence. We present a case of primary angiosarcoma that developed in a patient who had bilateral breast reduction surgery in the past.

## 1. Introduction

Angiosarcoma of the breast is a rare malignancy of endothelial cell origin and may occur spontaneously without any inciting event or as a result of chronic lymphedema or radiation. Primary angiosarcomas can be insidious or mistaken for other disease processes and have a high risk of metastatic disease. Obtaining a thorough history and physical exam, appropriate imaging, and an adequate biopsy for diagnosis is essential for early recognition and treatment. Surgery is the primary therapeutic intervention for breast angiosarcomas with radiation and chemotherapy as adjuvant treatment. Herein, we report a unique case of primary angiosarcoma that developed in what was initially mistaken for an infected keloidal after bilateral breast reduction surgery.

## 2. Case Report

**Figure 1:**
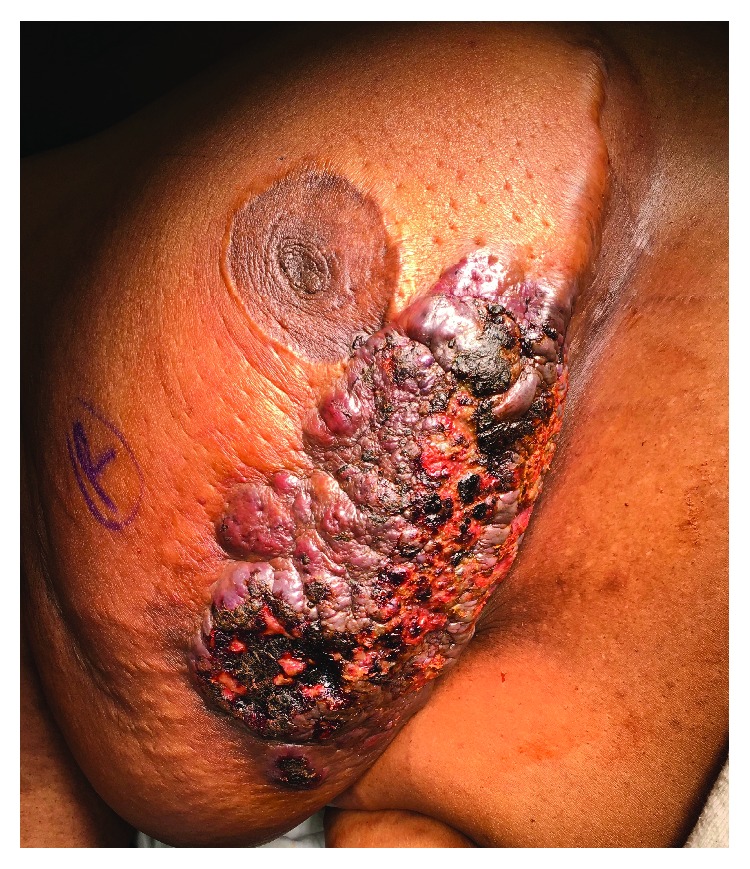


The patient is a 50-year-old African American female with a history of bilateral breast reduction twelve years ago, iron deficiency anemia, and obesity, who presented to the surgeon's office complaining of tenderness of her right breast. The patient reported that recently she had been developing keloids along the scar of the right breast with some areas having a blue hue; her left breast was unremarkable. She noticed that after wearing a sports bra there was increased pressure and abrasions to the keloid, leading to cellulitis and edema. She was previously treated with two courses of antibiotics for what was presumed to be an infected keloidal scar of her right breast but with minimal improvement. On exam, she had a large 10 cm diameter keloidal region on the inferior and lateral aspect of the right breast with edema and cellulitis. The keloidal area had no palpable fluctuance; she exhibited no nipple discharge or palpable adenopathy of the right axilla (Figure 1).

The patient had a benign-appearing mammogram 8 months prior, and all of her screening mammograms since her breast reduction have been without signs of malignancy. Another mammogram was ordered but was not performed due to patient discomfort. An ultrasound of the breast was preformed and suggested marked edema and skin thickening suggestive of infection but no definitive fluid collection or underlying suspicious mass was observed.

The patient underwent a right breast partial mastectomy for cosmesis and resection of the infected keloidal area. Intraoperatively, the mass was highly vascular, firm, but not fixed to the chest wall. Postoperatively, the pathology revealed a high-grade primary angiosarcoma of the breast with negative margins.

Patient underwent a computed tomography of the chest, abdomen, and pelvis, which did not show any evidence of gross metastatic disease. The patient then underwent completion mastectomy and scheduled for adjuvant chemotherapy with combination gemcitabine and Taxotere, followed by radiation.

## 3. Pathology

### 3.1. Materials and Methods

**Figure 2:**
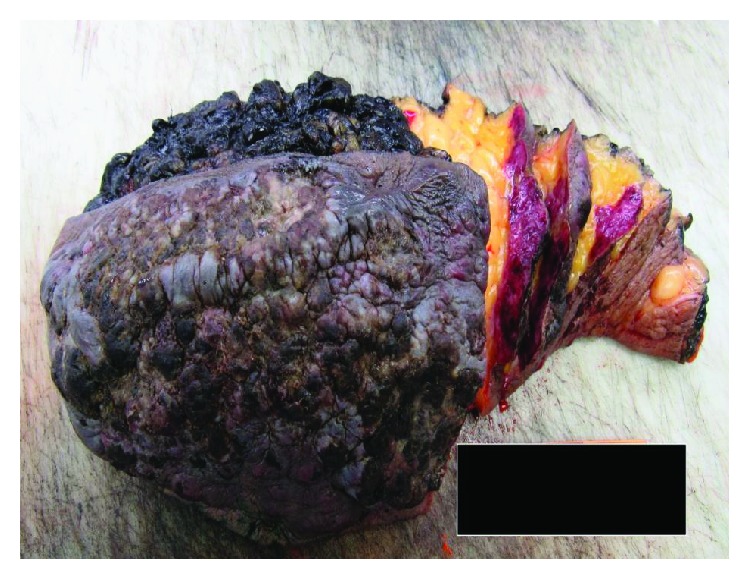
Blue-purple firm multinodular lesion measuring 20 cm × 11 cm on surface of skin, with possible ulceration and scab formation. Black ink designating inferior aspect of partial mastectomy.

**Figure 3:**
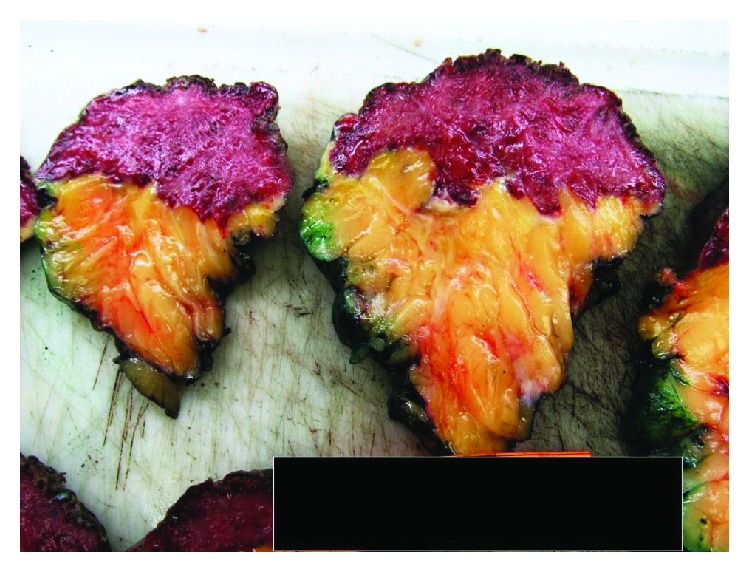
Cut sections of skin and underlying breast parenchyma. The lesion shows a red trabeculated/reticulated cut surface that infiltrates into the fibrofatty breast parenchyma in a lobulated fashion.

A gross depiction of the mass following right partial mastectomy is shown in (Figure 2) with cut sections shown in (Figure 3). The mass measured 20 × 11 cm and had a multinodular appearance with a bluish-purple hue with areas of ulceration.

**Figure 4:**
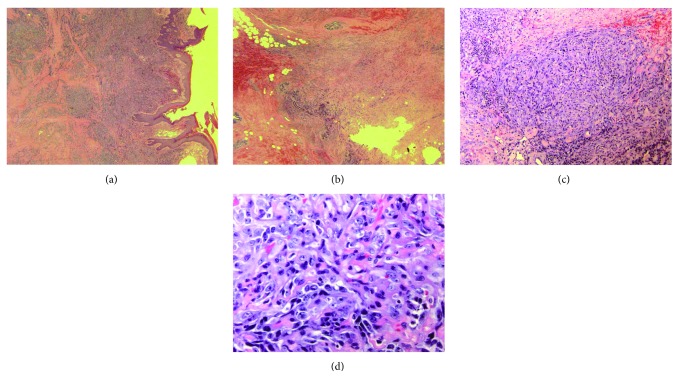
(a) 2x H&E stain. Skin with hyperkeratosis and underlying dermis containing nodular areas of poorly formed vascular spaces. (b) 2x H&E stain. Breast tissue composed of glands and adipose tissue with invasion by angiosarcoma. (c) 10x H&E stain. Infiltrating atypical spindle cells in a nodular pattern making poorly formed vascular channels with extravasated red blood cells. (d) 40x H&E stain. Atypical spindle endothelial cells with pleomorphic, large irregular nuclei, with prominent nucleoli, and multiple mitotic figures. There are red blood cells inside poorly formed vascular lumens as well as surrounding the spindle cells.

**Figure 5:**
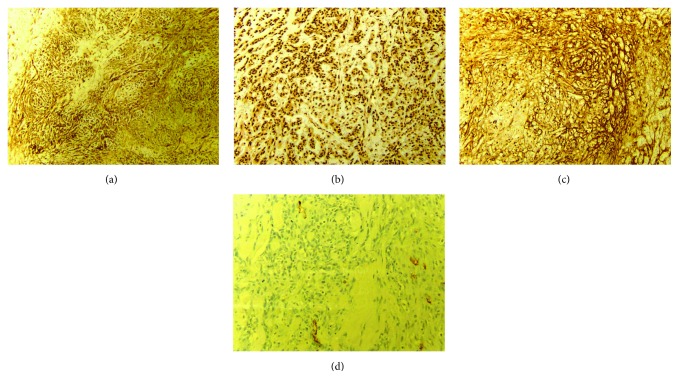
(a) 4x CD31 immunohistochemical stain. Positive membranous CD31 highlighting malignant endothelial cells. (b) 10x ERG immunohistochemical stain. Positive nuclear ERG immunostain in the malignant endothelial cells. (c) 10x factor VIII immunohistochemical stain. Positive cytoplasmic stain of malignant endothelial cells. (d) 20x CD34 immunohistochemical stain. Negative CD34, another membranous endothelial cell marker, failing to stain the malignant cells. Pancytokeratin immunostain was also negative.

Light microscopic images were obtained using standard H&E staining protocols on paraffin-embedded tissue sections. Microscopic examination revealed that the tumor was composed of high-grade atypical spindle endothelial cells with poorly formed vascular spaces (Figures 4(a)–4(d)). Immunochemistry analysis using antibodies to CD31, nuclear ERG, and factor VIII was positive, while analysis using CD34 and pancytokeratin was negative (Figures 5(a)–5(d)).

## 4. Discussion

Angiosarcoma is a rare breast tumor of endothelial cell origin accounting for less than 1% of all breast malignancies [1]. They are categorized as either primary or secondary. Primary angiosarcomas occur sporadically without an inciting factor. Treatment for primary angiosarcomas of the breast includes total mastectomy or modified radical mastectomy (MRM) depending on depth of invasion and involved margins [2]. Due to primarily hematogenous spread, axillary lymph node dissection is not typically performed. Surgery is followed by chemotherapy; options include anthracycline-based chemotherapy with ifosfamide or taxane-based chemotherapy in combination with gemcitabine showing marginal efficacy in metastatic disease [3–6]. In addition, the role of radiation therapy has shown promise in local regional control but not consistently proven to improve survival [7–9].

Secondary angiosarcomas occur after surgery and radiation therapy. The median time of occurrence of angiosarcoma after radiation therapy is approximately 6 years [10–12]. These tumors tend to be very aggressive with high rates of local recurrence and metastases. They generally require a mastectomy or MRM with consideration for radiation therapy. In addition, there is a potential survival benefit for taxane-based chemotherapy for recurrent or metastatic tumors.

Due to the fast, aggressive nature of these tumors, awareness is necessary of an association with Kasabach-Merritt syndrome. This occurs when there is consumptive coagulopathy and bleeding into a quickly growing angiosarcoma [13]. The occult nature of breast angiosarcomas requires close surveillance; there have been multiple case reports of primary angiosarcoma of the breast occurring in pregnant patients, one of which had metastatic disease to the contralateral breast [14, 15].

The standard of care and evaluation of such breast maladies includes an adequate biopsy. However, punch biopsies and core needle biopsies of vascular tumors of the breast tend to be challenging for pathologists, often requiring a larger incisional biopsy or wide local excision for definitive diagnosis and operative planning [16–18].

Primary angiosarcomas of the breast have been histologically classified into well differentiated (grade I), intermediate (grade II), or poorly differentiated (grade III) [19]. Most studies show correlation of long-term prognosis with higher tumor grade. Overall 5-year survival after surgery and chemoradiation for primary angiosarcoma of the breast is approximately 70–79% for grade I and II lesions and 15–30% for grade III lesions [20–22].

Interestingly, ultrasonography performed on patients with angiosarcomas tends to suggest an inflammatory or infectious process such as an abscess rather than neoplasia. Both ultrasound and mammogram findings have been nonspecific with regard to angiosarcomas of the breast as is in our case report; however, on MRI, T2 hypointense foci with rapid early arterial enhancement and washout have shown greater specificity to represent tumor foci [23].

We present what we believe to be a very rare and unusual case report of a primary angiosarcoma in a breast that had undergone breast reduction surgery twelve years prior to presentation and no history of radiation exposure. Angiosarcomas need to be considered in evaluation of breast masses in patients with a history of breast reduction surgery, seemingly unresolved breast infection, and with negative radiological imaging.
